# Supplementary material for: Factor XI in Carriers of Antiphospholipid Antibodies: Elevated Levels Associated with Symptomatic Thrombotic Cases, While Low Levels Linked to Asymptomatic Cases
Source: Int J Mol Sci. 2023 Nov 13;24(22):16270. doi: 10.3390/ijms242216270 (PMC10670960; doi:10.3390/ijms242216270)
Supplement: Supplementary file 1 [file ijms-24-16270-s001.zip › ijms-2676313-supplementary.pdf]

Supplementary table 1. Antiphospholipid antibodies of aPL patients according to the FXI:C levels.

	**FXI:C <70% (N= 13)**	**FXI:C >150% (N= 18)**	***p***
**LA N (%)**	10 (76.9)	13 (72.2)	1.00
**LA only N(%)**	6 (46.2)	6 (33.3)	0.470
**aCL**			
IgM N (%)	2 (15.4)	3 (16.7)	0.924
IgG N (%)	3 (23.1)	3 (16.7)	0.656
IgM and/or IgG N(%)	4 (30.8)	7 (38.9)	0.641
IgM and IgG N(%)	1 (7.7)	0 (0)	0.232
**aCL only N(%)**	0 (0)	1 (5.6)	0.388
**a*β*2GP1**			
**IgM N(%)**	3 (25)	1 (5.6)	0.32
**IgG N(%)**	4 (30.8)	6 (33.3)	1.00
IgM and/or IgG N (%)	4 (30.8)	6 (33.3)	1.00
IgM and IgG N(%)	2 (16.7)	1 (5.6)	
**a*β*2GP1 only N(%)**	0 (0)	2 (11.1)	0.214
Nº aPL			
**1 N (%)**	6 (46.2)	8 (44.4)	1.00
**2 N (%)**	0 (0)	3 (16.7)	0.35
**3 N (%)**	4 (30.8)	4 (22.2)	0.90
Thrombotic aPL profile			
**Low N (%)**	3 (23.1)	5 (27.8)	1.00
**High N (%)**	10 (76.9)	13 (72.2)	1.00

Abbreviations: antiphospholipid autoantibodies (aPL); anti β2 glycoprotein B1 (aβ2GP1); anti cardiolipin (aCL); Lupus anticoagulant (LA).

Supplementary table 2. Correlation analysis between coagulant activity of FXI and anticardiolipin and antiβ2GP1 antibody titre.

	**FXI:C (SS)**		**FXI:C (SFX)**	
	Correlation coeficient	**p**	Correlation coeficient	**p**
aβ2GP1 IgM	-0.23	**0.002**	-0.21	**0.005**
aβ2GP1 IgG	-0.22	**0.002**	-0.16	**0.031**
aCL IgM	-0.15	**0.034**	-0.14	0.057
aCL IgG	-0.197	**0.006**	-0.13	0.081

Abbreviations: anti β2 glycoprotein B1 (aβ2GP1); anti cardiolipin (aCL).

Supplementary table 3. Genetic defects, FXI:C and type of FXI deficiency detected in 4 cases with congenital FXI deficiency identified in this study.

**Patient**	**Group**	**cDNA variant**	**Protein variant**	**FXI:C**	**Type of deficiency**	**Reference HGMD**
**P1**	APS	c.403G>T	p.Glu135Ter	50%	CRM-	CM890042
**P2**	AaPL	c.403G>T	p.Glu135Ter	49%	CRM-	CM890042
**P3**	AaPL	c.802C>T	p.Arg268Cys	44%	CRM-	CM035499
**P4**	HC	c.1327C>T	p.Arg443Cys	23%	CRM-	CM062624

Abbreviations: Asymptomatic carriers of antiphospholipid antibodies (AaPL); Healthy controls (HC); Primary antiphospholipid syndrome (APS).
